# Supplementary material for: Impact of the COVID-19 pandemic on women in the workplace in the Middle East and North Africa: A scoping review protocol
Source: PLoS One. 2025 Feb 27;20(2):e0312037. doi: 10.1371/journal.pone.0312037 (PMC11867327; doi:10.1371/journal.pone.0312037)
Supplement: S1 Appendix — (DOCX) [file pone.0312037.s002.docx]

**Appendix 1. Search Strategy**

**Medline**

| 1 | coronaviridae/ or coronavirus/ or exp Coronavirus Infections/ or exp Betacoronavirus/ |
| --- | --- |
| 2 | (pneumonia/ or pneumonia, viral/ or exp Viruses/) and (exp Disease Outbreaks/ or exp Epidemiology/ or Epidemiology.fs.) |
| 3 | (coronavirus/ or betacoronavirus/ or coronavirus infections/) and (disease outbreaks/ or epidemics/ or pandemics/) |
| 4 | (betacoronavir* or beta-coronavir* or coronavir* or COVID* or "corona vir*").ti,ab. |
| 5 | 1 or 2 or 3 or 4 |
| 6 | limit 5 to yr="2019 -Current" |
| 7 | exp COVID-19/ or exp COVID-19 Testing/ or COVID-19 Vaccines/ or SARS-CoV-2/ |
| 8 | (COVID19 or COVID-19 or nCov* or "CoV 2" or CoV2 or 2019nCov* or 2019-nCov* or 19nCoV* or 19-nCov* or 2019Cov* or 2019-Cov* or HCoV* or "novel CoV").ti,ab. |
| 9 | ((novel or new or "19" or "2019" or wuhan or huanan or hubei or china or chinese) adj3 (coronavir* or "corona vir*" or betacoronavir* or "beta corona*" or CoV or COVID* or "severe acute respiratory" or SARS* or pneumonia)).ti,ab. |
| 10 | ((Coronavir* or "corona vir*" or betacoronavir* or "beta corona*" or cov*) adj3 (pandemic* or epidemic* or outbreak* or crisis or crises or disease*)).ti,ab. |
| 11 | (SARS2 or SARS-CoV-2 or SARSCOV-2 or SARS-COV2 or SARSCOV2 or (SARS adj2 (coronavir* or "corona vir*")) or "severe acute respiratory syndrome corona*").ti,ab. |
| 12 | (longCOVID* or "long COVID*" or postCOVID* or "post COVID*" or postcoronavir* or "postcorona vir*" or "post corona vir*" or "post coronavir*" or postSARS* or "wuhan virus" or "wuhan pneumonia").ti,ab. |
| 13 | 7 or 8 or 9 or 10 or 11 or 12 |
| 14 | 6 or 13 |
| 15 | exp "Quality of Life"/ |
| 16 | Health/ or Mental Health/ or exp physical fitness/ or reproductive health/ or sexual health/ |
| 17 | Fear/ or Anxiety/ or exp Violence/ or Psychological Distress/ |
| 18 | Mood Disorders/ or Depression/ |
| 19 | (wellbeing or "well being" or wellness or depress* or health* or welfare or anxiet* or anxious* or violence or violent* or stress* or distress* or fear* or (mood adj2 disorder*) or (qualit* adj2 life)).ti,ab. |
| 20 | 15 or 16 or 17 or 18 or 19 |
| 21 | employment/ or career mobility/ or employment, supported/ or return to work/ or teleworking/ or workplace/ |
| 22 | work/ or work-life balance/ or workload/ or work engagement/ or work performance/ |
| 23 | exp Occupations/ |
| 24 | exp Workforce/ |
| 25 | Salaries and Fringe Benefits/ |
| 26 | (work* or job or jobs or employ* or profession* or occupation* or trade or trades or career* or labor* or labour* or endeavor* or endeavour* or vocation* or salary or salaries or wage or wages or telework* or telecommut* or pay or pays or payment* or income*).ti,ab. |
| 27 | 21 or 22 or 23 or 24 or 25 or 26 |
| 28 | women/ or dentists, women/ or physicians, women/ or pregnant women/ or women, working/ or mothers/ |
| 29 | (woman or women or female* or girl or girls or mother* or maternal*).ti,ab. |
| 30 | 28 or 29 |
| 31 | algeria/ or egypt/ or libya/ or morocco/ or tunisia/ or djibouti/ or somalia/ or sudan/ or iraq/ or jordan/ or lebanon/ or syria/ or yemen/ |
| 32 | (iraq* or jordan* or lebanon or lebanese or yemen* or algeria* or egypt* or libya* or morocc* or tunis* or sudan* or palestin* or gaza or "west bank" or "east* jerusalem" or djibouti* or djibuti* or somal* or syria*).ti,ab. |
| 33 | 31 or 32 |
| 34 | 14 and 20 and 27 and 30 and 33 |
| 35 | exp Women's Health/ |
| 36 | 14 and 27 and 33 and 35 |
| 37 | occupational health/ |
| 38 | 14 and 30 and 33 and 37 |
| 39 | 34 or 36 or 38 |
| 40 | Limit 39 to yr="2020 -Current" |
