# Supplementary material for: Impact of the COVID-19 pandemic on women in the workplace in the Middle East and North Africa: A scoping review protocol
Source: PLoS One. 2025 Feb 27;20(2):e0312037. doi: 10.1371/journal.pone.0312037 (PMC11867327; doi:10.1371/journal.pone.0312037)
Supplement: S2 Appendix — (DOCX) [file pone.0312037.s003.docx]

**Appendix 2. Data Extraction Instrument**

| Study Information | | | | Study Design and Methodology | | | | | | | |
| --- | --- | --- | --- | --- | --- | --- | --- | --- | --- | --- | --- |
| Study title | Author | Year | Journal | Qualitative vs Quantitative vs Mixed | Study design | Country | Study setting | Sample size | Sampling method | Data collection method | Data analysis method |
|  |  |  |  |  |  |  |  |  |  |  |  |

| Participant Characteristics | | | Study Outcomes/Results | | Interventions | | Quality Assessment | | Conclusions and Implications | | | |  |  |  |  |  |
| --- | --- | --- | --- | --- | --- | --- | --- | --- | --- | --- | --- | --- | --- | --- | --- | --- | --- |
| Occupation | Sector | Formal or informal worker | Limitations | | Measures taken at the workplace | | Limitations | | Key conclusions of the study | | Implications for policy or practice | |  |  |  |  |  |
|  |  |  |  |  |  |  | |  | |  | |  | |  |  |  |  |
