# Supplementary material for: Impact of the COVID-19 pandemic on women in the workplace in the Middle East and North Africa: A scoping review protocol
Source: PLoS One. 2025 Feb 27;20(2):e0312037. doi: 10.1371/journal.pone.0312037 (PMC11867327; doi:10.1371/journal.pone.0312037)
Supplement: Attachment — Submitted filename: Peer Review of the Manuscript.docx [file pone.0312037.s004.docx]

Peer Review of the Manuscript: Impact of the COVID-19 Pandemic on Women in the Workplace in the Middle East and North Africa: A Scoping Review Protocol

**Summary Of review:**

The protocol is describing the study design for a study that the authors intend to undertake synthesizing the evidence on occupational health outcomes for women workers in the MENA region and their impact on the health (mental and physical) of the women. This is a very important research question that will enable the identification of regional priorities for occupational health for women in MENA, an important barrier to participation in the workforce.

**Background and context:** The general context of the study is well explained, outlining the global and regional evidence on women occupational health and the related challenges arising from COVID 19 at a global level. It also presents the general landscape of female participation in the workforce and the related health challenges. Given the fact that COVID 19 in general exacerbated many health-related aspects, the decision to conduct the scoping review is indeed valid.

**In terms of objectives of the study:** The primary outcome of the study is the mental and physical health wellbeing of women workers. The objectives could be nuanced a bit given that it is informing the research methods of a larger project. It would be good to include an objective on the state of evidence itself. The types of studies that have been done and the quality therein. It should also identify the gaps in evidence if any. The authors state in the inclusion criteria that “Studies conducted among populations within the countries of interest on the basis of ethnicity, religion, or country of origin will not be excluded, as the review aims to examine experiences of diverse populations of women workers, including migrant workers and refugees. This kind of nuance should be brought formally upfront as part of the specific objectives and secondary outcomes of interest in the review.

**The exclusion criteria** are well spelt out and comprehensive enough to establish the state of the evidence relevant to the primary objectives and secondary objectives including the impact on the diversity in the population.

**The time range on the study:** Since some cases of post COVID syndrome/Long COVID have been known to appear months after the case, it might be prudent to have a longer period to capture any study that highlights cases of mental and physical health challenges that arose after the pandemic ended.

The methods adopted for study selection, data extraction and process are aligned with PRISMA guidelines. The use of COVIDENCE is appropriate for dealing with duplicates.

**The Data analysis plan** is also clear and concise.

**Overall Recommendation: Consider for publication with minor changes summarized below. I need not review.**

- Elaborate the objectives of the study and nuance them to reflect the entire scope of what the study intends to achieve as highlighted above.
- Reconsider the time limits of the study to reflect the possibility of post Covid syndrome and studies that have documented these outcomes.
