# Supplementary material for: Impact of the COVID-19 pandemic on women in the workplace in the Middle East and North Africa: A scoping review protocol
Source: PLoS One. 2025 Feb 27;20(2):e0312037. doi: 10.1371/journal.pone.0312037 (PMC11867327; doi:10.1371/journal.pone.0312037)
Supplement: Attachment — Submitted filename: Comment.docx [file pone.0312037.s005.docx]

This manuscript titled “Impact of the COVID-19 Pandemic on Women in the Workplace in the Middle East and North Africa: A Scoping Review Protocol “ is well written and its methodology well detailed. It should prove useful for policy in the MENA region and add useful data and insight to research in a much needed area.

I however ,have listed few points that the writers need to resolve below.

1) Notably, women constitute an sizable share of the healthcare and education sectors in MENA, two sectors disproportionately impacted by the COVID-19 pandemic (5, 6).

What is this share? -elaborate with numbers and statistics for better clarity.

Grammar correction for the underlined highlighted words above- a sizeable share

2)The review will consider studies conducted in all MENA countries which meet World Bank criteria as low- or middleincome, including Algeria, Egypt, Iraq, Jordan, Lebanon, Libya, Morocco, Palestine, Sudan, Tunisia, and Yemen.

List out all the countries eligible for the study expressly= do not use including…

3)Ethical consideration. Ethical approval is not required for this review of the published, peer-reviewed literature.

Submission to ethic review is important and gives confidence that a local oversight role is available and applied . I would suggest submission for ethics consideration; an official documented waiver can then be granted . This waiver is quoted in lieu of ethical approval.
